# Temporal Trends in Mortality Related to Stroke and Atrial Fibrillation in the United States: A 21‐Year Retrospective Analysis of CDC‐WONDER Database

**DOI:** 10.1002/clc.70058

**Published:** 2024-12-16

**Authors:** Owais Ahmad, Hanzala Ahmed Farooqi, Isra Ahmed, Adeena Jamil, Rayyan Nabi, Irfan Ullah, Abdul Wali Khan, Raheel Ahmed, Mahboob Alam, Bernardo Cortese, Mamas A. Mamas

**Affiliations:** ^1^ Islamic International Medical College Riphah International University Islamabad Pakistan; ^2^ Aga Khan University Karachi Pakistan; ^3^ Department of Medicine Dow International Medical College Karachi Pakistan; ^4^ Department of Internal Medicine Khyber Teaching Hospital Peshawar Pakistan; ^5^ Department of Internal Medicine University of Missouri ‐ Kansas City Kansas City Missouri USA; ^6^ National Heart and Lung Institute Imperial College London London UK; ^7^ Division of Cardiology Baylor College of Medicine Houston Texas USA; ^8^ Harrington Heart & Vascular Institute University Hospitals Cleveland Medical Center Cleveland Ohio USA; ^9^ Fondazione Ricerca e Innovazione Cardiovascolare Milano Italy; ^10^ Keele Cardiovascular Research Group Keele University Stoke on Trent UK; ^11^ National Institute for Health and Care Research (NIHR) Birmingham Biomedical Research Centre Birmingham UK

**Keywords:** atrial fibrillation, CDC wonder, mortality, stroke, temporal trends

## Abstract

**Aims:**

Atrial Fibrillation (AF) is one of the most strongly associated risk factors for stroke. Our study aims to analyze changes in mortality from 1999 to 2020 in patients with AF and stroke.

**Methods:**

Using the Centre for Disease Control and Prevention Wide‐Ranging Online Data for Epidemiologic Research (CDC‐WONDER), we retrospectively analyzed annual age‐adjusted mortality rates (AAMR) per million from 1999 to 2020 in stroke patients with AF. Temporal trends were analyzed, and Annual Percentage Change (APC) was calculated using the JoinPoint regression model across variations in demographics (sex, race) and regional subgroups.

**Results:**

Around 490 000 deaths were reported between 1999 and 2020 from stroke and AF across the 25–85+ age group. AAMR initially decreased until 2008 (APC = –0.9), followed by an increase till 2020 (APC = 1.1). Women had a higher AAMR than men throughout the years. Non‐Hispanic white patients had a marginally higher AAMR than all other races and ethnicities. The highest AAMR was observed in the western region. States like Vermont, Oregon, Washington, Alaska, Minnesota, and West Virginia were in the top 90th percentile, while Nevada, Louisiana, Florida, New York, New Mexico, and Arizona were in the bottom 10th percentile. Nonmetropolitan areas had consistently higher AAMRs throughout the 2 decades.

**Conclusion:**

An overall rise in mortality has been observed in stroke and AF patients, with a greater surge in 2019. The need for healthcare policy changes, especially in areas with high mortality and awareness of healthier lifestyle factors, can be an essential preventative measure to help mitigate growing mortality rates.

## 1 Introduction

Atrial fibrillation (AF) is the most prevalent cardiac arrhythmia which is associated with a multitude of risk factors, including advancing age, smoking, alcohol consumption, body mass index (BMI), hypertension, valvular heart disease, heart failure, and myocardial infarction [1]. With an aging population, the incidence and prevalence of AF is increasing, thereby presenting a significant global health challenge and burden on healthcare systems.

AF is one of the most important modifiable risk factors linked with acute stroke [2]. AF is the underlying cause for approximately 15% of all strokes in the United States, 36% of strokes in people over 80, and up to 20% of cryptogenic strokes—that is, more than 100 000–125 000 embolic strokes annually, more than one‐fifth of which result in mortality [3, 4]. While studies utilizing data from CDC WONDER (Centers for Disease Control and Prevention Wide‐Ranging Online Data for Epidemiologic Research) database have been identified discussing mortality trends in patients labeled as a case of Stroke, no study has specifically describing temporal trends in patients of stroke that were suffering with AF. Given the strong correlation that exists between these two diseases, it is imperative to analyze AF and stroke related mortality trends. This will allow a better understanding of the detrimental effects of AF and stroke across various population groups; ultimately, allowing clinicians to tailor their treatment strategies effectively to improve patient outcomes.

Therefore, we evaluated the geographical and demographic variations in patients labeled as mortality due to stroke and AF among adults in the United States who were 25 years of age or older, from 1999 to 2020.

## 2 Methods

### 2.1 Study Setting and Population

In this retrospective study, we utilized publicly available death certificate data retrieved from the CDC WONDER (Centers for Disease Control and Prevention Wide‐Ranging Online Data for Epidemiologic Research) database. We analyzed death certificate data from 1999 to 2020 for Stroke‐related mortality in individuals with AF aged 25 years or older, using the following International Statistical Classification of Diseases and Related Health Problems‐10th Revision (ICD‐10) codes: I60.x, I61.x, I63.x, I64, I69.0, I69.1, I69.3, I69.4, I48. These ICD‐10 codes have been used previously to identify stroke and AF in administrative databases [2, 4]. The Multiple Cause‐of‐Death Public Use records were analyzed, and deaths related to stroke and AF were identified. This was based on the presence of AF and stroke listed anywhere on the death certificate, whether as a contributing factor or an underlying cause of death. The study followed the Strengthening the Reporting of Observational Studies in Epidemiology (STROBE) reporting guidelines and did not require approval from the local institutional review board [5].

### 2.2 Data Abstraction

State, year, region, population size, demographics, site of death, and urban‐rural classification were among the abstracted data. The demographics included the place of death, race/ethnicity, sex, and age. The data were further organized based on the place of death. In the course of this investigation, all death locations observed in the database were categorized into three groups: Hospital or Nursing Home (Inpatient, Outpatient or Emergency Room, Dead on Arrival, Unknown Status, Long‐Term Care Facility), Home or Hospice (Decedent's residence, Hospice facility), and Other (Other, Place of death unknown). The following categories of race and ethnicity were used: non‐Hispanic (NH) White, NH Black or African American, Hispanic or Latino, NH American Indian or Alaskan Native, NH Asian or Pacific Islander. The National Center for Health Statistics Urban‐Rural Classification Scheme was used to assess the population by urban (large metropolitan area [population > 1 million], medium/small metropolitan area [population 50 000–999 999]) and rural (population < 50 000) counties per the 2013 US census classification [3, 6]. The U.S. Census Bureau's division of regions into the Northeast, Midwest, South, and West was used to classify the regions [6].

### 2.3 Statistical Analysis

Crude and age adjusted mortality rates (AAMRs) were calculated per 100 000 population from 1999 to 2020 by year, sex, race/ethnicity, state, and urban‐rural status with 95% confidence intervals (CIs). This provided a better understanding and allowed us to analyze national trends in AF and stroke related mortality in the US. The indirect method of estimating age‐standardized mortality rates used the US Census of 2000 as the standard population [7]. Crude mortality rates were computed by dividing the overall number of stroke‐related deaths among AF patients by the corresponding U.S. population for that year. To evaluate national yearly trends in stroke‐related mortality in AF patients, the Joinpoint Regression Program (Joinpoint V 4.9.0.0, National Cancer Institute) was used to determine the annual percent change (APC) with 95% confidence interval (CI) in AAMR [8, 9]. This allows identification of significant changes in AAMR over time by fitting log‐linear regression models where temporal variation occurred. APCs were considered increasing or decreasing if the slope describing the change in mortality was significantly different from zero using 2‐tailed *t*‐testing. A value of *p* < 0.05 was considered statistically significant.

## 3 Results

**Figure 1:**
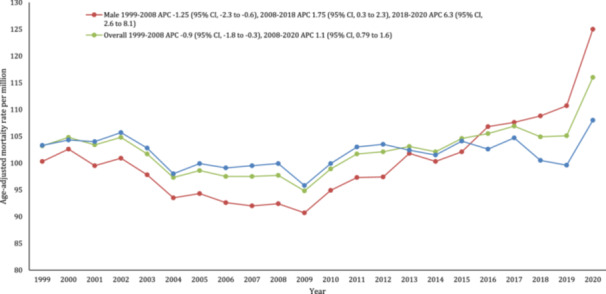
Trends in overall and sex‐stratified age‐adjusted AF and stroke‐related mortality rates among adults aged 25+ in the United States, 1999–2020. *Indicates that the annual percentage change (APC) is significantly different from zero at α = 0.05.

A total of 488 469 atrial fibrillation and stroke‐related deaths among patients aged 25 years or older occurred between 1999 and 2020, constituting 11.1% of stroke‐related mortalities wherein individuals had co‐existing atrial fibrillation. Out of these 45% of the deaths occurred in medical facility‐inpatient, followed by nursing homes/long‐term care (29.7%), decedent's home (15.4%), hospice facility (5.8%), and others (Supporting Information S1: Figure 1, Supporting Information S1: Table 1).

### 3.1 Annual Patterns in Mortality

The AAMR for stroke and AF‐related deaths was 103.2 per million in 1999 and 116.0 per million in 2020. The overall AAMR declined from 1999 to 2008 (APC: −0.9; 95% CI: −1.8 to −0.4) followed by an increase from 2008 to 2020 (APC: 1.1; 95% CI: −0.1 to 1.6) (Figure 1, Supporting Information S1: Table 2).

### 3.2 Mortality Patterns by Demographic Groups

#### 3.2.1 Gender

Females had consistently higher AAMRs than males from 1999 to 2015, thereafter higher AAMRS were reported in males from 2016 to 2020. In 1999, the AAMR for females was 103.3 (95% CI: 101.4–105.1), which decreased to 99.1 in 2006 (APC: −0.9; 95% CI: −3.5 to −0.1), followed by an increase to 108.0 in 2020 (APC:0.4; 95% CI: 0.1–1.5). Similarly, the AAMR for males in 1999 was 100.3 (95% CI: 97.8–102.9), which steadily decreased to 92.4 in 2008 (APC: −1.3; 95% CI: −2.3 to −0.6), followed by a steady increase till 2018 (APC: 1.8; 95% CI: 0.3–2.3). After this, there was a sharp increase till 2020 (APC: 6.3; 95% CI: 2.6–8.1). (Figure 1, Supprting Information S1: Table 2).

### 3.3 Race/Ethnicity

**Figure 2:**
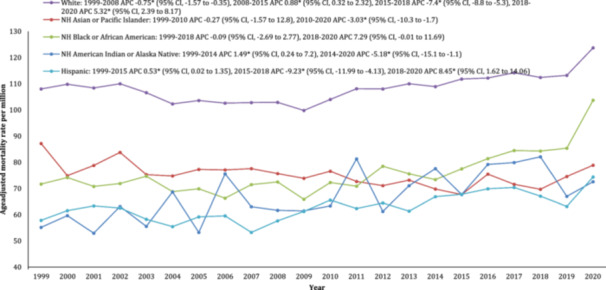
Trends in age‐adjusted stroke‐related mortality rates stratified by race/ethnicity among adults aged 25–85 years in the United States, 1999 to 2020. *Indicates that the annual percentage change (APC) is significantly different from zero at α = 0.05.

When stratified by race/ethnicity, AAMR per million was highest among NH White patients (108.8) followed by NH Black or African American (76.9), NH Asian or Pacific Islander (74.4), NH American Indian or Alaskan Native (68.5), and Hispanic or Latino (64.3) respectively. The AAMR for NH white declined until 2008 (APC: −0.9; 95% CI: −1.7 to −0.3) followed by an increase till 2020 (APC: 1.3; 95% CI: 1.0–1.7). The AAMR for the NH Black or African American population steadily declined from 1999 to 2009 (APC: −0.5; 95% CI: −4.5 to 0.8), followed by an increase till 2018 (APC: 2.1; 95% CI: −0.1 to 3.6) and finally a steep increase till 2020 (APC: 9.9; 95% CI: 3.6–13.6). A similar trend was noted in NH Asians or Pacific Islanders with a steep increase from 2018 to 2020 (APC: 6.3; 95% CI: 0.8–9.3). An initial decline in mortality was observed for all races except NH American Indians or Alaskan Natives and Hispanics or Latinos which increased from 1999 to 2020 (Figure 2, Supprting Information S1: Table 3).

### 3.4 Mortality Patterns Stratified by Geographic Region

**Figure 3:**
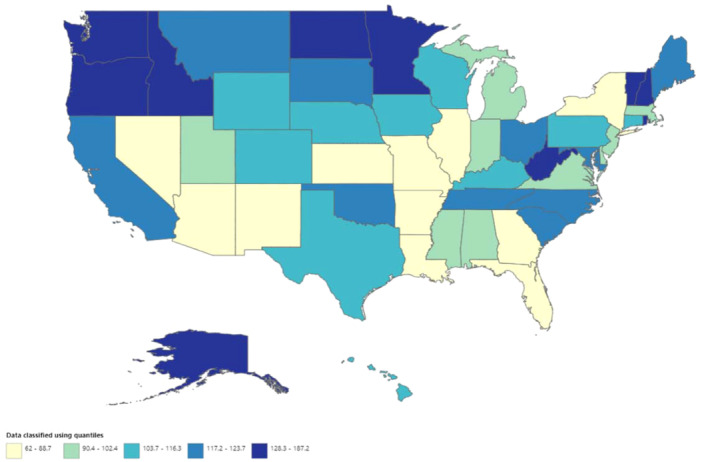
State‐level stroke‐related age‐adjusted mortality rates among AF adults aged 25–85+ years in the United States, 1999–2020.

**Figure 4:**
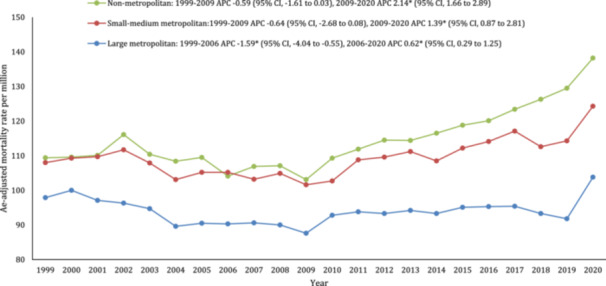
Trends in age‐adjusted stroke‐related mortality rates among adults aged 25–85+ years stratified by urban‐rural classification in the United States, 1999–2020. *Indicates that the annual percentage change (APC) is significantly different from zero at α = 0.05.

A significant difference in AAMR was observed in different states, with the AAMRs ranging from 62 in Nevada to 187.2 in Vermont. States that fell into the top 90th percentile were Vermont, Oregon, Washington, Alaska, Minnesota, and West Virginia, while the states that fell into the lower 10th percentile were Nevada, Louisiana, Florida, New York, New Mexico, Arizona (Figure 3, Supporting Information S1: Table 4). On average, throughout the study period, the highest AAMR was observed in the Western region (120.4), followed by the Midwestern region (103.8), Southern (95.8), and Northeast (94.4) regions (Supporting Information S1: Table 5). Nonmetropolitan areas had consistently higher stroke and AF‐related AAMRs than large metropolitan areas from 1999 to 2020, with overall AAMRs of 115.1 and 94.2, respectively. The small‐medium metropolitan areas lay in between with an overall AAMR of 109.9. Following an initial decline, an increase in mortality rates was noted in both nonmetropolitan and small‐medium metropolitan areas from 2009 to 2020 (APC 1.40 and 2.41 respectively) (Figure 4, Supporting Information S1: Table 6).

## 4 Discussion

In this nationwide study, we report several important findings. First, we describe an overall increase of 72.3% in the occurrence of stroke and AF‐related mortality among patients during the period spanning 1999–2000. Across this period, we report an initial decrease in AAMR from 1999 to 2008, with an inflection point and a subsequent increase in mortality rate from 2008 to 2020, which is sharpest from 2019 to 2020. This pattern was noted in both men and women and in all three classes of urbanization. Second, from 1999 to 2015, women consistently exhibited higher age‐adjusted stroke mortality rates than men, with a reversal in trends observed from 2016 to 2020, indicating a noteworthy shift in gender‐specific patterns. Third, we observed relatively high mortality rates in the NH‐White racial group as compared to all other ethnicities whereas Hispanics had the lowest AAMRs throughout the 21 years. Fourth, our findings show the highest mortality rates due to AF and stroke in the western region, followed by the midwestern, southern, and northeast regions. Moreover, in the broader context, nonmetropolitan regions exhibited the highest AAMR, while the large metropolitan areas did not show as great of an increase in mortality through the study period.

Before 2010, studies indicated a decreasing trend in stroke mortality within the general population [9, 10, 11]. However, subsequent temporal analyses [10, 11, 12] have identified the unfavorable changes of slowing, stagnating, and reversing the previously declining death rates. The observed changes in stroke mortality trends may be ascribed to shifts in specific risk determinants, notably the escalating prevalence of obesity, diabetes, suboptimal dietary practices, and sedentary behaviors over the recent decade [11, 13]. Particularly noteworthy is the discernible surge of these risk factors among the younger demographic, thereby contributing to an upward trajectory in stroke hospitalization rates among young adults [10, 11]. DeLago et. al also described this rising trend as a result of the underutilization of anti‐coagulation in AF, stating that in the US National Cardiovascular Data registry for patients with AF, fewer than half of high‐risk patients (CHA2DS2‐VACs ≥ 4) were being managed with anticoagulants [14]. Untreated AF patients had a 2.1 times greater risk of recurrent stroke and a 2.4 times greater risk of severe recurrent stroke. This is in contrast with the relationship witnessed in a study with a similar study design in Sweden conducted from 2001 to 2020. This study depicted an overall decline in stroke and AF‐related deaths, particularly after 2010, around which time novel oral anticoagulants (NOACs) were introduced to the Swedish market [15]. Additionally, an increase in compliance with lipid‐lowering drugs and the use of secondary preventive drugs [16], along with the global rise in obesity prevalence being significantly less in Sweden and other European countries compared to the USA has resulted in an overall decrease in AF‐stroke mortality.

Although our study reflects higher age‐adjusted stroke mortality rates and AF in women initially, the latter half of the study shows a reversal of the trend. Previously, it had been reported that there are higher stroke and stroke‐related mortality risks in women. Past studies referencing the CHA2DS2‐VASc score and risk stratification of stroke secondary to atrial fibrillation have demonstrated that female sex, in the context of atrial fibrillation, is an independent risk factor for stroke‐related mortality [17, 18, 19]. In addition, a multicenter Chinese study by Li et al. emphasized women's elevated risk of more debilitating strokes and stroke‐related deaths up to twofold, which persists even after adjusting for potential confounders. Additionally, a Canadian study spanning 2007–2016 found that the prevalence of AF rises with age, disproportionately affecting women due to their longer life expectancy. Furthermore, previously women with AF have been reported to encounter disparities in treatment, including under‐treatment, under‐dosing, and reduced time in the therapeutic range on warfarin relative to men [17, 20]. However, over the past half‐decade, the worldwide incidence of strokes has risen for both genders, with a more pronounced increase noted among men [13, 19]. These trends may be partly explained by changes in healthcare advocacy over the recent years such as women's greater health awareness, proactive health‐seeking behaviors, and improved access to primary prevention measures.

Overall, all races observed an increase in AAMRs by 2020 as compared to what was initially observed in 1999, with the highest rates in the NH White racial group. Our results are in contrast with the follow‐up study of 15 000 patients by Jared W. et al. that observed a higher risk of stroke as an outcome of AF patients among Black patients which was almost double that observed in White Americans [21]. Similarly, the CDC reported that NH‐Black men had a 70% greater chance of dying from stroke as compared to NH‐White, yet our data demonstrated significantly higher AAMRs among NH White patients. This may be due to the high prevalence of AF among NH‐White patients which is more than twice when compared to all other racial groups and accounts for 8% of the total White population [22]. Interestingly, our results also reported the lowest AAMR among Hispanics despite their population being more exposed to the related risk factors. This has also been previously reported in the literature as the “Hispanic paradox” [23]. Further investigation by researchers on a global scale is imperative to unveil the mystery and acquire further insights into plausible explanations. Nevertheless, the observation of racial disparities in mortality underscores the necessity for a multidisciplinary approach to establish health equity within diverse, culturally tailored institutions.

Although our findings show the highest mortality rates due to AF and stroke in the western region, the region‐wide mortality distribution has been noted to be the greatest in the south region followed by the west, then the midwest, and lastly the north [13, 14, 15]. This mortality cluster confirms past findings regarding higher stroke mortality being geographically concentrated in the southeast (relative risk 1.3–1.5) versus the rest of the country, known as the “stroke belt” [24] (30). This phenomenon was first documented in 1965 and describes an 8‐state region comprising of Alabama, Arkansas, Georgia, Louisiana, Mississippi, North Carolina, South Carolina, and Tennessee [25]. Yang et al reported a 4.2% rise in stroke death rates annually from 2013 to 2015 in the Southern census region versus a 0.6% increase in the western region [11]. These hot spots in the geographical distribution may be explained partially by differences in the prevalence of stroke risk factors, socieoeconomic factiors and the influence of selective migration. This can be exemplified by the lower median income and education, greater rates of unemployment; higher hospital admissions and emergency department visits per capita; increased incidences of obesity, diabetes mellitus, and hypertension; and significantly larger proportions of the black demographic [26]. Interestingly, when our data is organized by AAMR, the stroke and AF‐related mortality rate concentrations shift majorly to the western regions, followed by the southern region. This may be a result of a larger cluster of baby boomer populations in that region of the USA [14]. A reliance on state‐based approaches may overlook a lot of counties identified as hot spots of AF‐related stroke fatalities. Thus, further studies should focus on recognizing county‐level heterogenicity and reducing the change of misclassification bias which may be hindering a comprehensive understanding of the factors that may be influencing stroke mortality.

Following an initial decline, there was a subsequent upturn in mortality rates across all three categories, with nonmetropolitan regions experiencing the most pronounced increase from 2009 to 2020. The REGARDS study estimated the risk of stroke as a 30% increase between most rural and the most urban areas [27]. Similarly, our data showed an increasing difference between the two categories from 2009 onwards. The National Health and Nutrition Examination Survey has shown a greater prevalence of diagnosed hypertension, diabetes mellitus, and smoking in rural towns/cities, which could potentially be a consequence of the greater incidence of obesity in these areas as well as lower socioeconomic status [10, 27, 28]. These regional disparities may also be a result of inconsistencies in cardiology practice, limited access to quality health care, and the impact of state regulations on Medicaid. Between 2002 and 2015, the reduction in primary care providers was twice as pronounced in rural regions compared to their urban counterparts [28]. Despite the higher AAMRs, some studies have reported little significant interstate variance in the stroke case fatality between the two groups [24, 27, 29]. In a recent comprehensive survey encompassing stroke patients nationwide, 97.5% of rural patients were treated in a primary stroke center in contrast to 99.1% of the urban demographic. The use of online services such as Telestoke in the USA may also be assisting in mitigating the disparity between the two [27].

Our study had certain limitations; firstly the data retrieved relied solely on ICD‐10 codes assigned by WHO which may be prone to omissions or misrepresentations. Certain variables such as the socioeconomic status of patients were not assessed due to the absence of said data despite it being one of the critical determinants while assessing healthcare. Lastly, the database did not contain laboratory and clinical findings or the treatment history of patients which would have enabled us for a further comprehensive overview.

## 5 Conclusion

This investigation of Nationwide stroke‐related deaths among AF patients from 1999 to 2020 observed an initial drop followed by a large increase in mortality rates, notably after 2008. Gender disparities revealed women initially having higher stroke death rates, but this trend reversed after 2015, whilst racial differences suggested higher mortality rates for NH‐White persons. Regional patterns revealed greater death rates in the western and southern regions, with nonmetropolitan areas seeing the greatest rise. The observed patterns highlight the need for focused interventions that address risk factors, healthcare access, and regional disparities to reduce stroke‐related mortality and improve health equity across varied demographic groups and geographic areas.

## Ethics Statement

Since the data are publicly available and managed and deidentified by the Government of the USA, no ethical review was conducted.

## Conflicts of Interest

The authors declare no conflicts of interest.

## Supporting information

Supporting information.

## Data Availability

The data that support the findings of this study are openly available in CDC WONDER at https://wonder.cdc.gov/, reference number N/A. All study data are publicly available at https://wonder.cdc.gov.

## References

[1] G. Y. H. Lip , L. Fauchier , S. B. Freedman , et al., “Atrial Fibrillation,” Nature Reviews Disease Primers 2, no. 1 (2016): 16016. 10.1038/nrdp.2016.16 27159789

[2] M. J. O'Donnell , S. L. Chin , S. Rangarajan , et al., “Global and Regional Effects of Potentially Modifiable Risk Factors Associated With Acute Stroke in 32 Countries (INTERSTROKE): A Case‐Control Study,” The Lancet 388, no. 10046 (2016): 761–775. 10.1016/S0140-6736(16)30506-2 27431356

[3] R. B. Schnabel , X. Yin , P. Gona , et al., “50 Year Trends in Atrial Fibrillation Prevalence, Incidence, Risk Factors, and Mortality in the Framingham Heart Study: A Cohort Study,” The Lancet 386, no. 9989 (2015): 154–162. 10.1016/S0140-6736(14)61774-8 PMC4553037 25960110

[4] J. A. Reiffel , “Atrial Fibrillation and Stroke: Epidemiology,” The American Journal of Medicine 127, no. 4 (2014): e15–e16. 10.1016/j.amjmed.2013.06.002 24655742

[5] E. Von Elm , D. G. Altman , M. Egger , S. J. Pocock , P. C. Gøtzsche , and J. P. Vandenbroucke , “The Strengthening the Reporting of Observational Studies in Epidemiology (STROBE) Statement: Guidelines for Reporting Observational Studies,” Journal of Clinical Epidemiology 61, no. 4 (2008): 344–349. 18313558 10.1016/j.jclinepi.2007.11.008

[6] National Center for Health Statistics (U.S.) ., NCHS Urban‐Rural Classification Scheme for Counties (Hyattsville, Md: U.S. Dept. of Health and Human Services, Centers for Disease Control and Prevention, National Center for Health Statistics, 2012), 65.

[7] S. L. Murphy , J. Xu , K. D. Kochanek , E. Arias , and B. Tejada‐Vera , “Deaths: Final Data for 2018,” National Vital Statistics Reports: From the Centers for Disease Control and Prevention, National Center for Health Statistics, National Vital Statistics System 69, no. 13 (2021): 1–83. 33541516

[8] Joinpoint Regression Program [Internet]. [cited 2024 Oct 16]. Available from: https://surveillance.cancer.gov/joinpoint/.

[9] R. N. Anderson and H. M. Rosenberg , “Age Standardization of Death Rates: Implementation of the Year 2000 Standard,” National Vital Statistics System 47, no. 3 (1998): 1–16. 9796247

[10] R. W. Ariss , A. M. K. Minhas , J. Lang , et al., “Demographic and Regional Trends in Stroke‐Related Mortality in Young Adults in the United States, 1999 to 2019,” Journal of the American Heart Association 11, no. 18 (2022): e025903. 36073626 10.1161/JAHA.122.025903 PMC9683653

[11] Q. Yang , X. Tong , L. Schieb , et al., “Vital Signs: Recent Trends in Stroke Death Rates — United States, 2000–2015,” MMWR. Morbidity and Mortality Weekly Report 66, no. 35 (2017): 933–939. 28880858 10.15585/mmwr.mm6635e1 PMC5689041

[12] A. Gabet , C. Guenancia , G. Duloquin , V. Olié , and Y. Béjot , “Ischemic Stroke With Atrial Fibrillation: Characteristics and Time Trends 2006 to 2017 in the Dijon Stroke Registry,” Stroke 52, no. 6 (2021): 2077–2085. 33874745 10.1161/STROKEAHA.120.030812

[13] U. C. Mercy , K. Farhadi , A. S. Ogunsola , et al., “Revisiting Recent Trends in Stroke Death Rates, United States, 1999–2020,” Journal of the Neurological Sciences 451 (2023): 120724. 37421884 10.1016/j.jns.2023.120724

[14] A. J. DeLago , M. Essa , A. Ghajar , et al., “Incidence and Mortality Trends of Atrial Fibrillation/Atrial Flutter in the United States 1990 to 2017,” The American Journal of Cardiology 148 (2021): 78–83. 33640365 10.1016/j.amjcard.2021.02.014

[15] M. Ding , M. Ebeling , L. Ziegler , A. Wennberg , and K. Modig , “Time Trends in Atrial Fibrillation‐Related Stroke during 2001–2020 in Sweden: A Nationwide, Observational Study,” The Lancet Regional Health 28 (2023): 100596. 37180742 10.1016/j.lanepe.2023.100596 PMC10173271

[16] C. Drescher , F. Buchwald , T. Ullberg , M. Pihlsgård , B. Norrving , and J. Petersson , “Epidemiology of First and Recurrent Ischemic Stroke in Sweden 2010–2019: A Riksstroke Study,” Neuroepidemiology 56, no. 6 (2022): 433–442. 36223758 10.1159/000527373 PMC9945185

[17] R. C. Martin , W. S. Burgin , M. B. Schabath , et al., “Gender‐Specific Differences for Risk of Disability and Death in Atrial Fibrillation‐Related Stroke,” The American Journal of Cardiology 119, no. 2 (2017): 256–261. 27846983 10.1016/j.amjcard.2016.09.049

[18] Y. Tanaka , N. S. Shah , R. Passman , P. Greenland , D. M. Lloyd‐Jones , and S. S. Khan , “Trends in Cardiovascular Mortality Related to Atrial Fibrillation in the United States, 2011 to 2018,” Journal of the American Heart Association 10, no. 15 (2021): e020163. 34320819 10.1161/JAHA.120.020163 PMC8475678

[19] S. Barker‐Collo , D. A. Bennett , R. V. Krishnamurthi , et al., “Sex Differences in Stroke Incidence, Prevalence, Mortality and Disability‐Adjusted Life Years: Results from the Global Burden of Disease Study 2013,” Neuroepidemiology 45, no. 3 (2015): 203–214. 26505984 10.1159/000441103 PMC4632242

[20] A. C. Jönsson , J. Ek , and C. Kremer , “Outcome of Men and Women After Atrial Fibrillation and Stroke,” Acta Neurologica Scandinavica 132, no. 2 (2015): 125–131. 25649996 10.1111/ane.12366

[21] J. W. Magnani , F. L. Norby , S. K. Agarwal , et al., “Racial Differences in Atrial Fibrillation‐Related Cardiovascular Disease and Mortality: The Atherosclerosis Risk in Communities (ARIC) Study,” JAMA Cardiology 1, no. 4 (2016): 433. 27438320 10.1001/jamacardio.2016.1025 PMC5347977

[22] A. Yuh‐Jer Shen , R. Contreras , S. Sobnosky , et al., “Racial/Ethnic Differences in the Prevalence of Atrial Fibrillation Among Older Adults—A Cross‐Sectional Study,” Journal of the National Medical Association 102, no. 10 (2010): 906–914. 21053705 10.1016/s0027-9684(15)30709-4

[23] M. Cortes‐Bergoderi , K. Goel , M. H. Murad , et al., “Cardiovascular Mortality in Hispanics Compared to Non‐Hispanic Whites: A Systematic Review and Meta‐Analysis of the Hispanic Paradox,” European Journal of Internal Medicine 24, no. 8 (2013): 791–799. 24095273 10.1016/j.ejim.2013.09.003

[24] J. Koifman , R. Hall , S. Li , et al., “The Association Between Rural Residence and Stroke Care and Outcomes,” Journal of the Neurological Sciences 363 (2016): 16–20. 27000213 10.1016/j.jns.2016.02.019

[25] Geographic Variations in Stroke Incidence and Mortality Among Older Populations in Four US Communities | Stroke [Internet]. [cited 2024 Oct 16]. Available from: https://www.ahajournals.org/doi/10.1161/01.str.0000231453.98473.67. 10.1161/01.STR.0000231453.98473.67 16794205

[26] D. N. Karp , C. S. Wolff , D. J. Wiebe , C. C. Branas , B. G. Carr , and M. T. Mullen , “Reassessing the Stroke Belt: Using Small Area Spatial Statistics to Identify Clusters of High Stroke Mortality in the United States,” Stroke 47, no. 7 (2016): 1939–1942. 27197853 10.1161/STROKEAHA.116.012997 PMC4927355

[27] G. Howard , D. O. Kleindorfer , M. Cushman , et al., “Contributors to the Excess Stroke Mortality in Rural Areas in the United States,” Stroke 48, no. 7 (2017): 1773–1778. 28626048 10.1161/STROKEAHA.117.017089 PMC5502731

[28] T. J. Siddiqi , A. M. Khan Minhas , S. J. Greene , et al., “Trends in Heart Failure–Related Mortality Among Older Adults in the United States From 1999‐2019,” JACC: Heart Failure 10, no. 11 (2022): 851–859. 36328654 10.1016/j.jchf.2022.06.012

[29] D. J. Lanska and L. H. Kuller , “The Geography of Stroke Mortality in the United States and the Concept of a Stroke Belt,” Stroke 26, no. 7 (1995): 1145–1149. 7604404 10.1161/01.str.26.7.1145

