# Supplementary material for: Temporal Trends in Mortality Related to Stroke and Atrial Fibrillation in the United States: A 21‐Year Retrospective Analysis of CDC‐WONDER Database
Source: Clin Cardiol. 2024 Dec 16;47(12):e70058. doi: 10.1002/clc.70058 (PMC11648035; doi:10.1002/clc.70058)
Supplement: Supplementary file 1 — Supporting information. [file CLC-47-e70058-s001.docx]

# **Temporal trends in mortality related to Stroke and Atrial Fibrillation in the United States: a 21-year retrospective Analysis of CDC-WONDER database**

# **Supplementary Table 1:** Absolute number of stroke-related deaths among Afib patients aged 25-85+ years stratified by place of death in the United States, 1999-2020

| **Year** | **Decedent's home** | **Hospice facility** | **Nursing home/long term care** | **Medical facility** | **Other** | **Place of death unknown** |
| --- | --- | --- | --- | --- | --- | --- |
| 1999 | 1675 | 36 | 6492 | 9563 | 353 | - |
| 2000 | 1909 | 75 | 6558 | 9728 | 421 | - |
| 2001 | 1957 | 268 | 6635 | 9601 | 494 | - |
| 2002 | 2167 | 493 | 6725 | 9678 | 605 | - |
| 2003 | 2213 | 685 | 6498 | 9435 | 688 | 58 |
| 2004 | 2162 | 885 | 6111 | 9231 | 707 | 66 |
| 2005 | 2279 | 894 | 6313 | 9413 | 666 | 67 |
| 2006 | 2349 | 1221 | 6188 | 9483 | 606 | 87 |
| 2007 | 2609 | 1482 | 6121 | 9506 | 652 | 52 |
| 2008 | 2622 | 1825 | 6000 | 9588 | 747 | 242 |
| 2009 | 2762 | 1811 | 5913 | 9229 | 750 | 331 |
| 2010 | 3090 | 2182 | 6179 | 9729 | 856 | 13 |
| 2011 | 3441 | 2582 | 6504 | 9985 | 956 | 13 |
| 2012 | 3672 | 2657 | 6447 | 10099 | 1012 | 16 |
| 2013 | 3881 | 2831 | 6709 | 10257 | 1122 | 17 |
| 2014 | 4090 | 2736 | 6862 | 10132 | 857 | 17 |
| 2015 | 4445 | 2925 | 6969 | 10495 | 788 | - |
| 2016 | 4789 | 2973 | 6837 | 10803 | 925 | - |
| 2017 | 4971 | 36 | 7183 | 11046 | 951 | - |
| 2018 | 5249 | 75 | 7159 | 11003 | 988 | - |
| 2019 | 5609 | 268 | 7235 | 11011 | 965 | - |
| 2020 | 7509 | 2973 | 7284 | 12044 | 1354 | - |
| Total | 75450 | 28561 | 144922 | 221059 | 17463 | 1014 |

## ‘–‘unavailable data on CDC WONDER database

## **Supplementary Table 2:** Absolute number of stroke-related deaths in Afib patients stratified by sex and race in the United States, 1999-2020

| **Year** | **Overall** | **Females** | **Males** | **NH-American Indian or Alaska Native** | **NH-Asian or Pacific Islander** | **NH-Black or African American** | **NH-White** | **Hispanic or Latino origin** |
| --- | --- | --- | --- | --- | --- | --- | --- | --- |
| 1999 | 18085 | 11800 | 6285 | 31 | 298 | 982 | 16330 | 407 |
| 2000 | 18620 | 12093 | 6527 | 37 | 270 | 1030 | 16793 | 454 |
| 2001 | 18688 | 12214 | 6474 | 35 | 306 | 994 | 16804 | 505 |
| 2002 | 19178 | 12512 | 6666 | 39 | 345 | 1015 | 17227 | 518 |
| 2003 | 18928 | 12324 | 6604 | 38 | 334 | 1075 | 16932 | 516 |
| 2004 | 18352 | 11878 | 6474 | 47 | 348 | 1008 | 16413 | 516 |
| 2005 | 19006 | 12322 | 6684 | 41 | 397 | 1044 | 16919 | 583 |
| 2006 | 19206 | 12436 | 6770 | 56 | 417 | 1020 | 17069 | 622 |
| 2007 | 19625 | 12722 | 6903 | 49 | 442 | 1123 | 17407 | 592 |
| 2008 | 20084 | 12972 | 7112 | 50 | 456 | 1169 | 17723 | 661 |
| 2009 | 19879 | 12675 | 7204 | 49 | 479 | 1101 | 17460 | 753 |
| 2010 | 21088 | 13425 | 7663 | 51 | 501 | 1228 | 18444 | 836 |
| 2011 | 22381 | 14181 | 8200 | 71 | 530 | 1256 | 19624 | 874 |
| 2012 | 23071 | 14594 | 8477 | 59 | 556 | 1443 | 20009 | 962 |
| 2013 | 23797 | 14651 | 9146 | 73 | 622 | 1432 | 20654 | 968 |
| 2014 | 24140 | 14830 | 9310 | 80 | 649 | 1451 | 20793 | 1113 |
| 2015 | 25288 | 15495 | 9793 | 81 | 665 | 1601 | 21641 | 1224 |
| 2016 | 26014 | 15501 | 10513 | 97 | 786 | 1745 | 22011 | 1319 |
| 2017 | 26983 | 16072 | 10911 | 103 | 804 | 1867 | 22746 | 1408 |
| 2018 | 27138 | 15735 | 11403 | 114 | 811 | 1928 | 22811 | 1427 |
| 2019 | 27749 | 15831 | 11918 | 103 | 932 | 2011 | 23287 | 1383 |
| 2020 | 31169 | 17326 | 13843 | 114 | 1046 | 2564 | 25685 | 1717 |
| Total | 488469 | 303589 | 184880 | 1418 | 11994 | 30087 | 42478 | 19358 |

NH- : Non-Hispanic

**Supplementary Table 3:** Age-Adjusted Mortality Rates per 1000,000 of stroke-related deaths in Afib patients stratified by sex and race in the United States, 1999-2020

| **Year** | **Overall** | **Females** | **Males** | **NH-American Indian or Alaska Native** | **NH-Asian or Pacific Islander** | **NH-Black or African American** | **NH-White** | **Hispanic**  **Or Latino origin** |
| --- | --- | --- | --- | --- | --- | --- | --- | --- |
| 1999 | 103.2 | 103.3 | 100.3 | 55.2 | 87.2 | 71.7 | 108 | 57.9 |
| 2000 | 104.8 | 104.3 | 102.6 | 59.7 | 74.9 | 74.2 | 109.8 | 61.6 |
| 2001 | 103.4 | 104 | 99.5 | 53 | 78.8 | 70.8 | 108.4 | 63.4 |
| 2002 | 104.8 | 105.7 | 100.9 | 63.2 | 83.8 | 71.9 | 110 | 62.6 |
| 2003 | 101.7 | 102.8 | 97.8 | 55.6 | 75.3 | 74.7 | 106.6 | 58.3 |
| 2004 | 97.3 | 98 | 93.5 | 68.7 | 74.8 | 68.8 | 102.3 | 55.5 |
| 2005 | 98.6 | 99.9 | 94.3 | 53.3 | 77.3 | 69.9 | 103.6 | 59.2 |
| 2006 | 97.5 | 99.1 | 92.6 | 75.6 | 77.1 | 66.3 | 102.6 | 59.6 |
| 2007 | 97.5 | 99.5 | 92 | 63.1 | 77.6 | 71.5 | 102.8 | 53.3 |
| 2008 | 97.7 | 99.9 | 92.4 | 61.7 | 75.7 | 72.5 | 102.9 | 57.7 |
| 2009 | 94.8 | 95.8 | 90.7 | 61.5 | 73.9 | 65.9 | 99.8 | 61.3 |
| 2010 | 98.9 | 99.9 | 94.9 | 63.4 | 76.6 | 72.3 | 104 | 65.6 |
| 2011 | 101.7 | 103 | 97.3 | 81.3 | 72.7 | 70.9 | 108.1 | 62.4 |
| 2012 | 102.1 | 103.5 | 97.4 | 61.3 | 71.1 | 78.5 | 108 | 64.5 |
| 2013 | 103.1 | 102.4 | 101.8 | 71.1 | 73.2 | 75.6 | 110 | 61.4 |
| 2014 | 102.1 | 101.5 | 100.3 | 77.6 | 69.8 | 73.4 | 108.9 | 66.9 |
| 2015 | 104.6 | 104.1 | 102.1 | 67.7 | 67.7 | 77.5 | 111.8 | 67.8 |
| 2016 | 105.5 | 102.6 | 106.8 | 79.2 | 75.5 | 81.4 | 112.2 | 69.9 |
| 2017 | 106.9 | 104.7 | 107.6 | 79.9 | 71.6 | 84.5 | 114.2 | 70.4 |
| 2018 | 104.9 | 100.5 | 108.8 | 82.1 | 69.7 | 84.3 | 112.4 | 67.1 |
| 2019 | 105.1 | 99.6 | 110.7 | 67 | 74.6 | 85.4 | 113.2 | 63.2 |
| 2020 | 116 | 108 | 125 | 72.6 | 78.9 | 103.7 | 123.7 | 74.4 |
| Total | 102.8 | 102.1 | 101.4 | 68.5 | 74.4 | 76.9 | 108.4 | 63.4 |

## **Supplementary Table 4:** Age-Adjusted Mortality Rates per 1000,000 of stroke-related deaths in Afib patients stratified by percentiles in the United States, 1999-2020

| **State** | **Deaths** | **Percentiles** | **Age Adjusted Rate** |
| --- | --- | --- | --- |
| Nevada | 2008 | 0 | 62 |
| Louisiana | 4152 | 2 | 65.7 |
| Florida | 26392 | 4 | 67.7 |
| New York | 23790 | 6 | 73.2 |
| New Mexico | 2185 | 8 | 73.8 |
| Arizona | 7400 | 10 | 75.5 |
| Georgia | 8534 | 12 | 76.8 |
| Illinois | 16204 | 14 | 82.5 |
| Kansas | 4009 | 16 | 82.9 |
| Arkansas | 4112 | 18 | 87.6 |
| Missouri | 8770 | 20 | 88.7 |
| District of Columbia | 775 | 22 | 90.4 |
| Michigan | 14696 | 24 | 91.4 |
| Delaware | 1335 | 26 | 93.6 |
| Massachusetts | 10970 | 28 | 94.1 |
| Mississippi | 4005 | 30 | 94.2 |
| Alabama | 6797 | 32 | 94.3 |
| Virginia | 10323 | 34 | 94.3 |
| New Jersey | 14060 | 36 | 96.1 |
| Utah | 2692 | 38 | 97 |
| Indiana | 10121 | 40 | 102.4 |
| Wyoming | 812 | 42 | 103.7 |
| Hawaii | 2512 | 44 | 104 |
| Connecticut | 6867 | 46 | 104.2 |
| Colorado | 6601 | 48 | 106.2 |
| Texas | 30792 | 50 | 106.4 |
| Pennsylvania | 26265 | 52 | 106.5 |
| Kentucky | 6978 | 54 | 109.4 |
| Iowa | 6569 | 56 | 110.3 |
| Wisconsin | 10855 | 58 | 111.6 |
| Nebraska | 3681 | 60 | 116.3 |
| Montana | 2002 | 62 | 117.2 |
| South Dakota | 1831 | 64 | 118.5 |
| Maryland | 9854 | 66 | 118.6 |
| North Carolina | 16240 | 68 | 120 |
| Oklahoma | 6856 | 70 | 120.2 |
| Maine | 3029 | 72 | 121.6 |
| South Carolina | 8186 | 74 | 121.9 |
| California | 63017 | 76 | 123.3 |
| Ohio | 23863 | 78 | 123.4 |
| Tennessee | 11486 | 80 | 123.7 |
| Idaho | 2810 | 82 | 128.3 |
| North Dakota | 1709 | 84 | 128.7 |
| New Hampshire | 2743 | 86 | 129.7 |
| Rhode Island | 2636 | 88 | 129.9 |
| West Virginia | 4410 | 90 | 133.4 |
| Minnesota | 12085 | 92 | 138.9 |
| Alaska | 721 | 94 | 139.8 |
| Washington | 16434 | 96 | 168.1 |
| Oregon | 11277 | 98 | 176.4 |
| Vermont | 2018 | 100 | 187.2 |

## **Supplementary Table 5:** Stroke-related age-adjusted mortality rates per 1000,000 among Afib patients aged 25-85 years stratified by census region in the United States, 1999-2020

| **Census Region** | **Year** | **Age Adjusted Rate** |
| --- | --- | --- |
| Northeast | 1999 | 99.6 |
| Northeast | 2000 | 100.6 |
| Northeast | 2001 | 100.4 |
| Northeast | 2002 | 98.1 |
| Northeast | 2003 | 94.6 |
| Northeast | 2004 | 91.4 |
| Northeast | 2005 | 89.1 |
| Northeast | 2006 | 88.2 |
| Northeast | 2007 | 89.2 |
| Northeast | 2008 | 87.7 |
| Northeast | 2009 | 85 |
| Northeast | 2010 | 92.6 |
| Northeast | 2011 | 94.7 |
| Northeast | 2012 | 97.5 |
| Northeast | 2013 | 96.7 |
| Northeast | 2014 | 97.3 |
| Northeast | 2015 | 97.4 |
| Northeast | 2016 | 98 |
| Northeast | 2017 | 95.6 |
| Northeast | 2018 | 92.5 |
| Northeast | 2019 | 90 |
| Northeast | 2020 | 100.1 |
| Northeast | Total | 94.4 |
| Midwest | 1999 | 102.1 |
| Midwest | 2000 | 103.6 |
| Midwest | 2001 | 99.9 |
| Midwest | 2002 | 105.3 |
| Midwest | 2003 | 99.2 |
| Midwest | 2004 | 96.4 |
| Midwest | 2005 | 99.8 |
| Midwest | 2006 | 96.5 |
| Midwest | 2007 | 99.3 |
| Midwest | 2008 | 100 |
| Midwest | 2009 | 94.8 |
| Midwest | 2010 | 98.8 |
| Midwest | 2011 | 102.6 |
| Midwest | 2012 | 103.7 |
| Midwest | 2013 | 104.5 |
| Midwest | 2014 | 104.4 |
| Midwest | 2015 | 106.5 |
| Midwest | 2016 | 107 |
| Midwest | 2017 | 109.6 |
| Midwest | 2018 | 108.5 |
| Midwest | 2019 | 109.9 |
| Midwest | 2020 | 124.4 |
| Midwest | Total | 103.8 |
| South | 1999 | 97.2 |
| South | 2000 | 98.6 |
| South | 2001 | 95.7 |
| South | 2002 | 99.4 |
| South | 2003 | 95.8 |
| South | 2004 | 89.8 |
| South | 2005 | 92.4 |
| South | 2006 | 91.4 |
| South | 2007 | 91.2 |
| South | 2008 | 89.6 |
| South | 2009 | 87.9 |
| South | 2010 | 91.7 |
| South | 2011 | 92.6 |
| South | 2012 | 93.7 |
| South | 2013 | 95 |
| South | 2014 | 93.3 |
| South | 2015 | 96.8 |
| South | 2016 | 97.1 |
| South | 2017 | 99.1 |
| South | 2018 | 99.6 |
| South | 2019 | 101.5 |
| South | 2020 | 111 |
| South | Total | 95.8 |
| West | 1999 | 118.9 |
| West | 2000 | 121.3 |
| West | 2001 | 124.5 |
| West | 2002 | 120.7 |
| West | 2003 | 122.6 |
| West | 2004 | 117 |
| West | 2005 | 117.2 |
| West | 2006 | 118.2 |
| West | 2007 | 114.1 |
| West | 2008 | 118.6 |
| West | 2009 | 115.3 |
| West | 2010 | 116.5 |
| West | 2011 | 122.2 |
| West | 2012 | 118.3 |
| West | 2013 | 120.1 |
| West | 2014 | 118.1 |
| West | 2015 | 121 |
| West | 2016 | 123.7 |
| West | 2017 | 127 |
| West | 2018 | 120.3 |
| West | 2019 | 119.1 |
| West | 2020 | 129.3 |
| West | Total | 120.4 |

## **Supplementary Table 6:** Stroke-related mortality in AFib patients age-adjusted mortality rates per 1000,000 among adults aged 25-85+ years stratified by urban-rural classification in the United States, 1999 to 2020

| **Year** | **Large-metropolitan** | **Medium-small metropolitan** | **Non-Metropolitan** |
| --- | --- | --- | --- |
| 1999 | 97.9 | 108 | 109.4 |
| 2000 | 100 | 109.3 | 109.6 |
| 2001 | 97.1 | 109.7 | 110.1 |
| 2002 | 96.3 | 111.7 | 116.1 |
| 2003 | 94.7 | 107.9 | 110.4 |
| 2004 | 89.6 | 103.1 | 108.4 |
| 2005 | 90.5 | 105.2 | 109.5 |
| 2006 | 90.3 | 105.2 | 104.1 |
| 2007 | 90.6 | 103.2 | 106.9 |
| 2008 | 90 | 104.9 | 107.1 |
| 2009 | 87.6 | 101.6 | 103.1 |
| 2010 | 92.8 | 102.7 | 109.3 |
| 2011 | 93.8 | 108.8 | 111.9 |
| 2012 | 93.3 | 109.6 | 114.5 |
| 2013 | 94.2 | 111.2 | 114.4 |
| 2014 | 93.3 | 108.5 | 116.5 |
| 2015 | 95.1 | 112.2 | 118.8 |
| 2016 | 95.3 | 114.1 | 120.1 |
| 2017 | 95.4 | 117.1 | 123.4 |
| 2018 | 93.3 | 112.6 | 126.3 |
| 2019 | 91.8 | 114.3 | 129.5 |
| 2020 | 103.8 | 124.3 | 138.2 |

## **Supplementary Figure 1:** Place of death distribution percentages
